# Supplementary material for: Commercial cannabis product testing: Fidelity to labels and regulations
Source: PLoS One. 2026 Apr 15;21(4):e0321832. doi: 10.1371/journal.pone.0321832 (PMC13082621; doi:10.1371/journal.pone.0321832)
Supplement: S1 Fig — Legend: The green bands on the graphs represent the allowable + /- 15% variation in concentration. (PDF) [file pone.0321832.s001.pdf]

Figure 1. Observed THC Product Concentration in the Context of Legally Allowable Variation

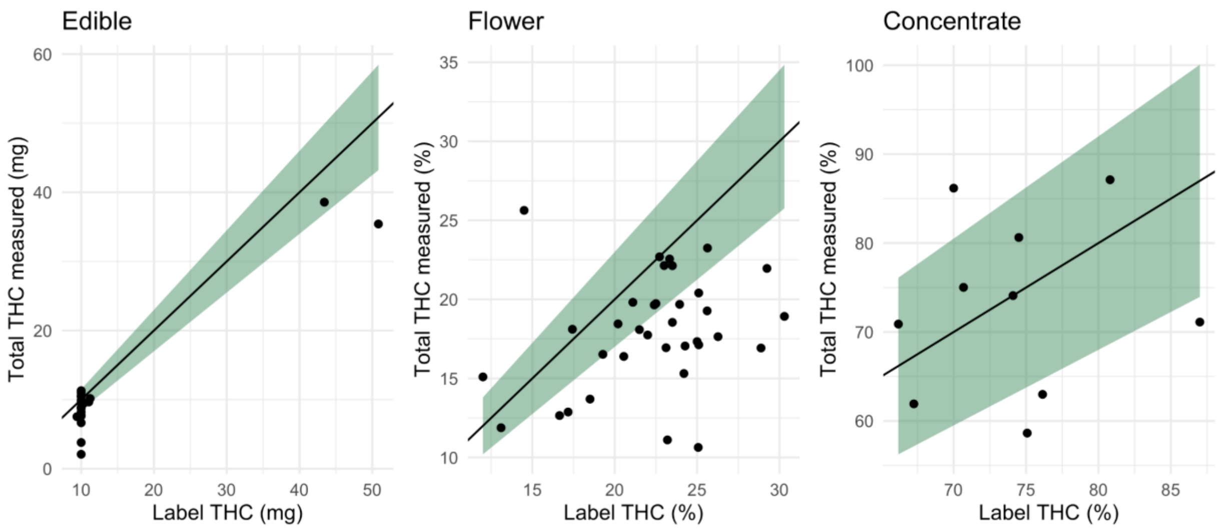

Note: The green bands on the graphs represent the allowable +/- 15% variation in concentration
