# Supplementary material for: Commercial cannabis product testing: Fidelity to labels and regulations
Source: PLoS One. 2026 Apr 15;21(4):e0321832. doi: 10.1371/journal.pone.0321832 (PMC13082621; doi:10.1371/journal.pone.0321832)
Supplement: S2 Fig — Legend: Samples were frozen after collection. (PDF) [file pone.0321832.s002.pdf]

Figure 2. Number of Days Between Purchasing Cannabis Product and Testing of THC Concentration by Difference between Labeled and Tested THC Concentration

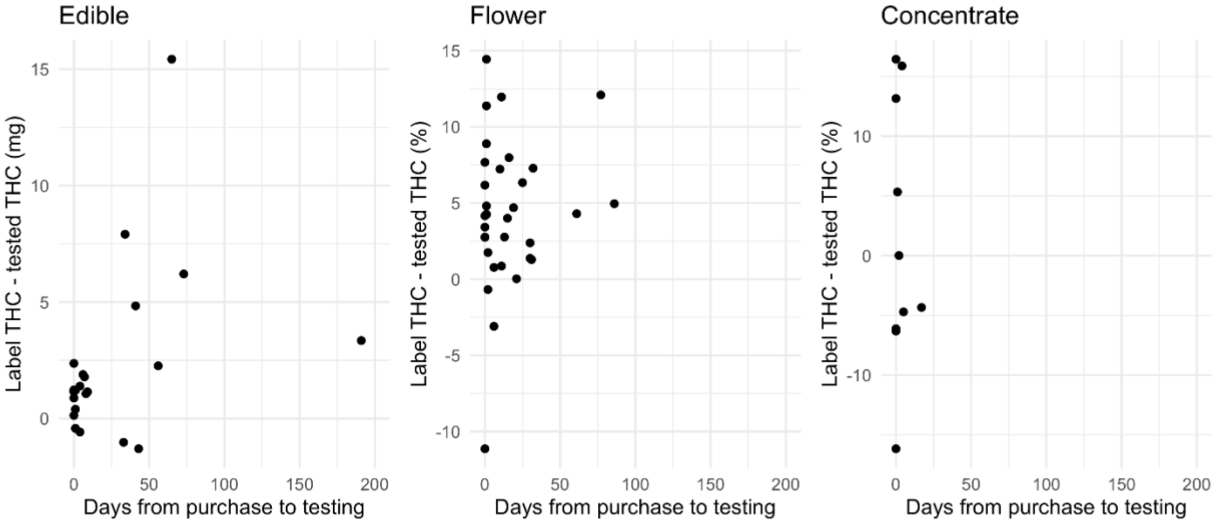

Note: Samples were frozen after collection
