# Supplementary material for: Commercial cannabis product testing: Fidelity to labels and regulations
Source: PLoS One. 2026 Apr 15;21(4):e0321832. doi: 10.1371/journal.pone.0321832 (PMC13082621; doi:10.1371/journal.pone.0321832)
Supplement: S1 Table — Legend: N = 7 participants did not have product type listed. (PDF) [file pone.0321832.s003.pdf]

Supplemental Table 1. THC concentration by edible subtype

Edible Type (n= 29)	n	Labeled THC Mean (min, max)	Observed THC Mean (min, max)
Candy	1	10.0 (10.0,10.0)	2.1 (2.1, 2.1)
Gummy	18	14.2 (9.4, 50.8)	12.2 (3.8, 38.6)
Powder	3	10.0 (10.0, 10.0)	7.5 (6.7, 8.2)

Note: N=7 participants did not have product type listed
