# Supplementary material for: Commercial cannabis product testing: Fidelity to labels and regulations
Source: PLoS One. 2026 Apr 15;21(4):e0321832. doi: 10.1371/journal.pone.0321832 (PMC13082621; doi:10.1371/journal.pone.0321832)
Supplement: S2 Table — (PDF) [file pone.0321832.s004.pdf]

Supplemental Table 2. Days from purchase of cannabis product to testing

Product Type (n= 74)	Mean	Standard Deviation	Min	Max
Edible (n=22)	26.23	43.7	0	191
Flower (n=33)	22.0	22.098	0	86
Concentrate (n=10)	5.3	5.3	0	17
